# P-618. Pooled Microbiological Outcomes from the Phase 3, Randomized OPTIC and OPTIC-2 Trials of Omadacycline vs Moxifloxacin in Community-acquired Bacterial Pneumonia

**DOI:** 10.1093/ofid/ofaf695.831

**Published:** 2026-01-11

**Authors:** Diane M Anastasiou, Surya Chitra, Alisa W Serio

**Affiliations:** Paratek Pharmaceuticals, Inc., King of Prussia, PA; Paratek Pharmaceuticals, Inc., King of Prussia, PA; Paratek Pharmaceuticals, Inc., King of Prussia, PA

## Abstract

**Background:**

Omadacycline (OMC) is an FDA-approved oral (PO) and intravenous (IV) treatment for adults with community-acquired bacterial pneumonia (CABP) and acute bacterial skin and skin-structure infections. We present pooled efficacy and microbiological response of OMC vs moxifloxacin (MOX) from two CABP phase 3 trials.

**Figure 1.**
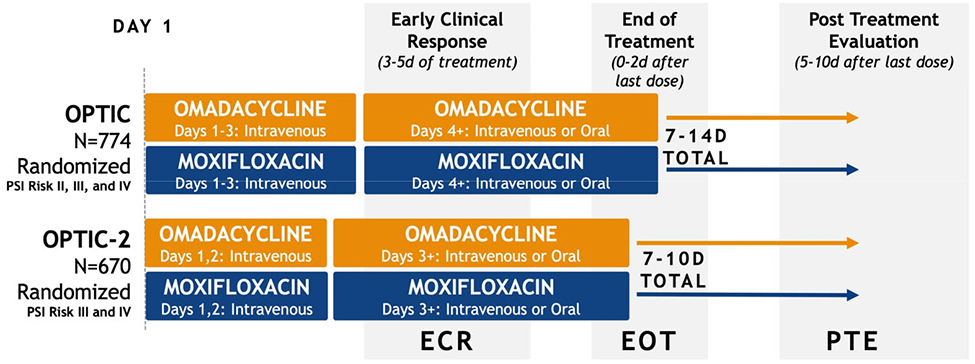
OPTIC and OPTIC-2 Study Designs. OPTIC: treatment was either OMC 100 mg IV q12h ×2 doses or 200 mg IV once per day on day 1, thereafter 100 mg IV q24h; or MOX 400 mg IV q24h for 3 days. OPTIC-2: OMC 100 mg IV q12h x2 doses or 200 mg IV once per day, or MOX 400 mg IV q24h on days 1-2. Thereafter, patients could transition to PO therapy (OMC 300 mg q24h or MOX 400 mg q24h) Total treatment duration was 7 to 14 days for OPTIC, and 7 to 10 days for OPTIC-2 with the option to extend to 14 days if the participant had bacteremia at baseline. Early clinical response (ECR; 72–120 h after first dose) was defined as survival, no receipt of rescue antibacterial therapy, and improvement in at least two of four symptoms (cough, sputum production, pleuritic chest pain, dyspnea) without deterioration in any of these symptoms. Secondary endpoints included investigator’s assessment of clinical response at end of treatment (EOT; 0–2 days after last dose) and post-therapy evaluation (PTE; 5–10 days after last dose), defined as survival with resolution of signs and symptoms of infection such that further antibacterial therapy was unnecessary.

**Table 1.**
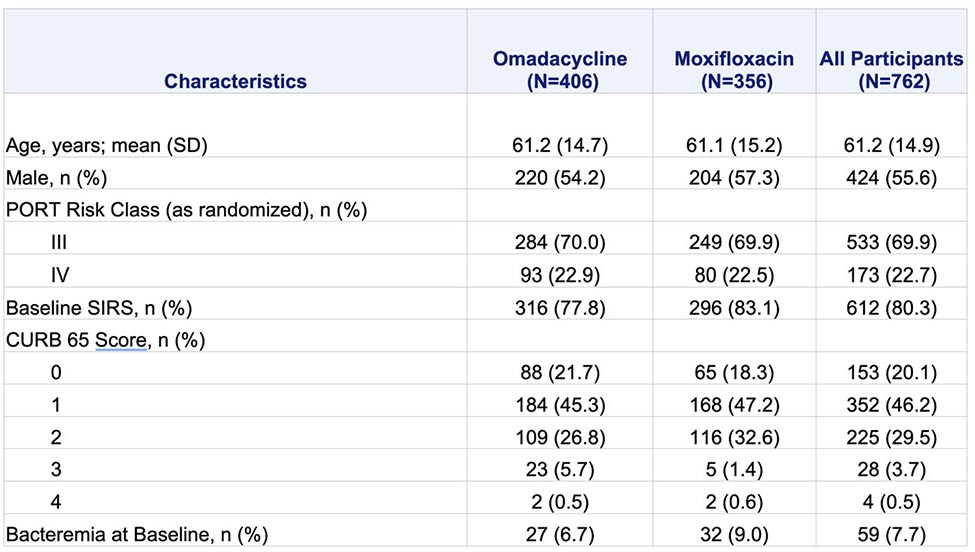
Demographic and Baseline Characteristics, Pooled microITT Population Microbiological intent-to-treat (microITT) population: all randomized participants for whom a baseline pathogen was identified. PORT = Pneumonia Outcomes Research Team, PORT Risk Class (actual) were based on PORT score (derived) from the case report form (CRF). CABP = community-acquired bacterial pneumonia; CURB-65 = confusion, uremia, respiratory rate, blood pressure, and age 65 or older; SD = standard deviation; SIRS = systemic inflammatory response syndrome SIRS criteria is defined as having 2 or more of the following 4 symptoms at baseline: temperature < 36°C or > 38°C (oral or oral equivalent), heart rate > 90 bpm, respiratory rate > 20 breaths/min, WBC < 4000 cells/mm3 or WBC > 12,000 cells/mm3, or bands > 10%.

**Methods:**

OPTIC (NCT02531438) and OPTIC-2 (NCT04779242) were phase 3, randomized, double-blind trials comparing OMC vs MOX for treatment of adults with CABP. OPTIC (global trial) included participants with Pneumonia Severity Index (PSI) class II, III, or IV; OPTIC-2 (Europe) included only PSI class III or IV. Initial treatment was OMC or MOX IV 1-3d, with the option to transition to PO after 3d (OPTIC) or 2d (OPTIC- 2); total treatment duration was 7–14d for OPTIC/OPTIC-2 (Figure 1). Primary endpoint, early clinical response (ECR; 72-120h after first dose), was: survival, no rescue antibacterial therapy, and improvement in ≥2 of 4 symptoms (cough, sputum production, pleuritic chest pain, dyspnea) without any deterioration. Key secondary endpoint was investigator’s assessment of clinical response at post-therapy evaluation (PTE; 5-10d after last dose): resolution or improvement of signs and symptoms, further antibacterial therapy not needed. Per-participant microbiological response at PTE was assessed.

**Table 2.**
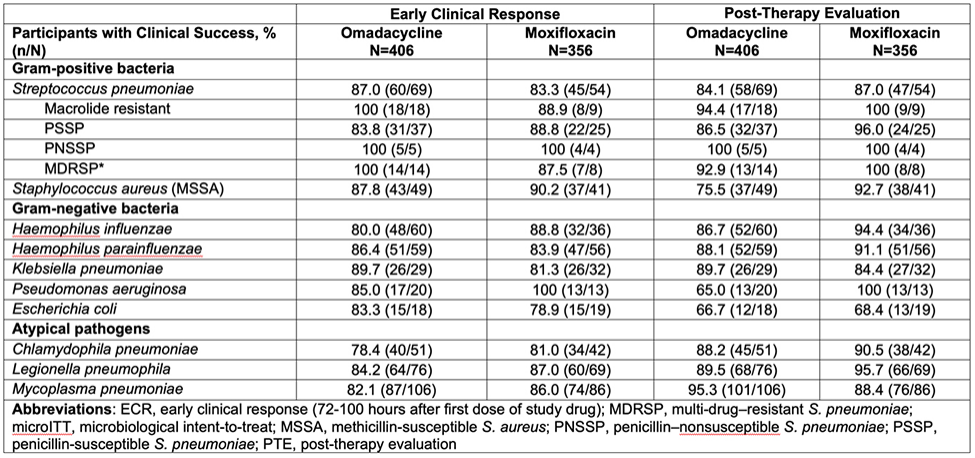
Clinical Success at ECR and PTE for Most Frequent Baseline Pathogens, Pooled microITT Population

Microbiological ITT (micro-ITT) population: all randomized participants who had at least one causative pathogen identified at screening. Participants with the same pathogen from blood culture, respiratory specimen culture, urinary antigen tests, and/or serology are counted only once for that pathogen. Participants with any combination of MDRSP, PNSSP, PSSP and Macrolide resistant are counted once in the overall tabulation for Streptococcus pneumoniae. Data are shown for baseline pathogens that were identified in ≥15 participants.

At baseline, a respiratory specimen (eg, expectorated or induced sputum, bronchoalveolar lavage) was collected for Gram staining and culture, blood cultures were collected, and urine was tested for antigens for L. pneumophila and S. pneumoniae. At baseline and PTE visits, blood was also collected for acute- and convalescent-phase serological testing for L. pneumophila, M. pneumoniae, and C. pneumoniae.

Early clinical response (ECR; 72–120 h after first dose) was defined as survival, no receipt of rescue antibacterial therapy, and improvement in at least two of four symptoms (cough, sputum production, pleuritic chest pain, dyspnea) without deterioration in any of these symptoms. Key secondary endpoint was investigator’s assessment of clinical response at post-therapy evaluation (PTE; 5–10 days after last dose), defined as survival with resolution of signs and symptoms of infection such that further antibacterial therapy was unnecessary.

*A pathogen was considered multidrug resistant if susceptibility testing shows resistance to at least one antibiotic within three or more different classes of antibiotics. For Streptococcus spp., isolates resistant to macrolides but susceptible to lincosamides and D-test positive (>4/0·5 µg/mL) were considered resistant to both macrolides and lincosamides.

**Table 3.**
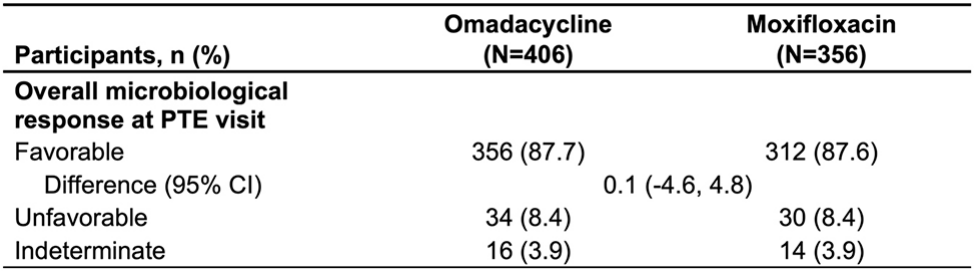
Per-participant Microbiological Response at PTE Visit 95% CI was constructed based on the Miettinen and Nurminen method with stratification. Percentages were based on the total number of participants at each visit in each treatment group. CI, confidence interval; microITT, microbiological intent-to-treat; PTE, post-therapy evaluation.

**Funding:**

This study was sponsored by Paratek Pharmaceuticals, Inc. This study described herein has been funded in whole or in part with federal funds from the U.S. Department of Health and Human Services (HHS), Administration for Strategic Preparedness and Response (ASPR), Biomedical Advanced Research and Development Authority (BARDA), under Contract No. 75A50120C00001. The contract and federal funding are not an endorsement of the study results, products, or company.

**Results:**

762 participants (microbiological intent-to-treat population [microITT]) were included across both studies (n=406 OMC, n=356 MOX; Table 1). The 5 most common baseline pathogens in the OMC and MOX groups, respectively, were *Mycoplasma pneumoniae* (26.1%, 24.2%) *Legionella pneumophila* (18.7%, 19.4%), *Streptococcus pneumoniae* (17.0%, 15.2%), *Haemophilus influenzae* (14.8%, 10.1%), and *H. parainfluenzae* (14.5%, 15.7%). Clinical success at ECR was ≥78% for the OMC group across the most common pathogens (Table 2). At PTE, clinical success by baseline pathogen was similarly high (Table 2). At PTE, the per-participant microbiological response was favorable for both groups: 87.7% (356/406) for OMC and 87.6% (312/356) for MOX (Table 3).

**Conclusion:**

Pooled OPTIC and OPTIC-2 data showed overall clinical success rates >86% for most baseline pathogens and favorable microbiological response rates at PTE for omadacycline in CABP.

**Disclosures:**

Diane M. Anastasiou, BS, Paratek Pharmaceuticals, Inc.: Employee Surya Chitra, PhD, Paratek Pharmaceuticals, Inc.: Advisor/Consultant Alisa W. Serio, PhD, Paratek Pharmaceuticals, Inc.: Employee

